# Effect of pH on antimicrobial activity of delafloxacin against *Escherichia coli* isogenic strains carrying diverse chromosomal and plasmid-mediated fluoroquinolone resistance mechanisms

**DOI:** 10.1128/spectrum.02338-25

**Published:** 2025-10-30

**Authors:** K. Alexandre, Marina Murillo-Torres, Fernando Docobo-Pérez, Álvaro Pascual, Jose Manuel Rodríguez-Martínez

**Affiliations:** 1 Department of Infectious Diseases, Univ Rouen Normandie, Université de Caen Normandie, INSERM, Normandie Univ, DYNAMICURE UMR 1311, CHU Rouen, Rouen, France; 2 Departamento de Microbiología, Facultad de Medicina, Universidad de Sevilla, Sevilla, Spain; 3 Instituto de Biomedicina de Sevilla (IBIS), Hospital Universitario Virgen Macarena/CSIC/Universidad de Sevilla, Sevilla, Spain; 4 Centro de Investigación Biomédica en Red en Enfermedades Infecciosas (CIBERINFEC), Instituto de Salud Carlos III 38176, Madrid, Spain; 5 Unidad Clínica de Enfermedades Infecciosas y Microbiología, Hospital Universitario Virgen Macarena, Sevilla, Spain; College of New Jersey, Ewing, New Jersey, USA

**Keywords:** PK/PD, microenvironment, pH, delafloxacin, fluoroquinolones, *Escherichia coli*, urinary tract infection

## Abstract

The aim of this study is to evaluate the pH-dependent antimicrobial activities of delafloxacin against isogenic *Escherichia coli* strains carrying diverse fluoroquinolone resistance mechanisms. Eighty-one isogenic *E. coli* strains derived from ATCC 25922 were tested, carrying various combinations of chromosomal mutations (*gyrA, parC*), efflux pump overexpression (*marR*), and plasmid-mediated quinolone resistance (PMQR) determinants (*qnrA1, qnrB1, qnrC, qnrD1, qnrS1, qepA2, aac(6')-Ib-cr*). Minimum inhibitory concentrations (MICs) were determined using broth microdilution at pH 7.3, 6.0, and 5.0. Time-killing assays were performed on three representative strains. Semi-mechanism-based mathematical modeling using Monolix quantified bactericidal activity through EC_50_ parameters. Ciprofloxacin MIC_90_ increased from 8 mg/L at pH 7.3 to 128 mg/L at pH 5.0, while delafloxacin MIC_90_ decreased from 64 mg/L to 16 mg/L. At physiological pH, ciprofloxacin demonstrated superior activity (median MIC: 1 mg/L vs 8 mg/L for delafloxacin, *P* < 0.001), while at pH 5.0, delafloxacin median MIC was 2 mg/L versus 64 mg/L for ciprofloxacin (*P* < 0.001). According to EUCAST breakpoints, susceptibility frequencies at pH 5.0 were 25.9% (21/81) for delafloxacin versus 2.5% (2/81) for ciprofloxacin (*P* < 0.001). Among PMQR, *qnr* genes led to 0% delafloxacin susceptibility across all conditions (0/150 strain-pH combinations), while *qepA2* (8/30 strain-pH combinations, 26.7%) and *aac(6')-Ib-cr* (16/30 strain-pH combinations, 53.3%) showed comparable susceptibility frequencies to isogenic strains not carrying PMQR (15/33 strain-pH combinations, 45.5%). Simulated clinical exposures against chromosomal mutant EC02 (GyrA-S83L) demonstrated that ciprofloxacin would fail at pH 5.0, while delafloxacin would maintain antimicrobial activity. Environmental pH deeply modulates fluoroquinolone activity and resistance mechanism impact. Delafloxacin demonstrates clinically relevant activity in acidic infection environments.

**IMPORTANCE:**

Fluoroquinolones face increasing resistance challenges, particularly in acidic infection sites, such as the urinary tract, abscesses, and biofilms, where pH can drop below 6.0. This study reveals a critical pH-dependent reversal in antimicrobial effectiveness between ciprofloxacin and delafloxacin against resistant *Escherichia coli*. While ciprofloxacin loses substantial activity in acidic conditions (90% of strains showing 16-fold minimum inhibitory concentration increases at pH 5.0), delafloxacin maintains and even enhances its antimicrobial potency, with susceptibility rates increasing from 25.9% to over 50% as pH decreases. Using comprehensive resistance profiling across 81 isogenic strains and pharmacokinetic modeling, we demonstrate that delafloxacin could overcome treatment failures in acidic infection sites where conventional fluoroquinolones fail. These findings could have immediate clinical implications for treating urinary tract infections, abscesses, and biofilm-associated infections, potentially expanding therapeutic options against multidrug-resistant bacteria in previously challenging anatomical sites.

## INTRODUCTION

Fluoroquinolones represent a critical class of broad-spectrum antibiotics widely used for treating serious Gram-negative infections. However, standard antimicrobial susceptibility testing (AST) is performed under optimal laboratory conditions that fail to reflect the hostile microenvironment encountered by bacteria at the site of infection (1). Clinical infection sites are characterized by nutrient limitation, oxidative stress, and notably, acidification to pH levels as low as 5.0–6.0 in abscesses, complicated urinary tract infections, and infected wounds (2). These environmental factors can significantly alter antibiotic efficacy, potentially leading to treatment failures despite apparent *in vitro* susceptibility.

The pH-dependent activity of fluoroquinolones varies significantly among different compounds. While zwitterionic fluoroquinolones like ciprofloxacin demonstrate reduced activity under acidic conditions, delafloxacin exhibits improved activity in acidic environments (3–6). However, systematic evaluation of how diverse fluoroquinolone resistance mechanisms affect pH-dependent activity patterns remains limited (7, 8). Of particular clinical importance, the impact of acidic pH on bactericidal activity has been insufficiently studied across different resistance backgrounds. This knowledge gap is clinically relevant given the increasing prevalence of multidrug-resistant Enterobacterales harboring complex combinations of chromosomal mutations and plasmid-mediated quinolone resistance (PMQR) determinants (9, 10).

We hypothesized that acidic pH could differentially modulate the impact of fluoroquinolone resistance mechanisms on antimicrobial activity. The primary objective was to comprehensively evaluate the pH-dependent activities of delafloxacin compared to ciprofloxacin against isogenic *Escherichia coli* strains harboring diverse fluoroquinolone resistance mechanisms.

## MATERIALS AND METHODS

### Bacterial strains, growth conditions, and antimicrobial agents

A total of 81 isogenic *E. coli* strains derived from the wild-type (WT) strain *E. coli* ATCC 25922 were used (Table S1). These isogenic strains carried various combinations of chromosomal mutations in quinolone resistance-determining regions (*gyrA, parC*), overexpression of efflux pumps (*marR*), and/or PMQR determinants (*qnrA1, qnrB1, qnrC, qnrD1, qnrS1, qepA2, aac(6')-Ib-cr*) as previously described (11). Briefly, *gyrA* and *parC* mutants were obtained by gene replacement, disruption of the *marR* gene was carried out using the Red helper pKOBEG system, and *qnr, qepA2,* and *aac(6')-Ib-cr* genes were amplified using designed primers and then cloned into the kanamycin-resistant pBK-CMV vector. The pBK-CMV cloning vectors containing genes of interest (*qnr* or *qepA2* or *aac(6')-lb-cr*) were then transformed into *E. coli* ATCC 25922 and its isogenic mutant strains. All constructions were confirmed by PCR.

Strains were cultured at 37°C in Mueller-Hinton broth (MHB) or Mueller-Hinton agar (MHA). Kanamycin (Sigma-Aldrich, Madrid, Spain) was added at 30 mg/L to ensure plasmid maintenance. Ciprofloxacin was obtained from Sigma-Aldrich and delafloxacin from MedChem Express (Sollentuna, Sweden). Stock solutions were prepared following Clinical Laboratory Standards Institute guidelines (M100 ED24).

### Antibiotic susceptibility testing

Minimum inhibitory concentrations (MICs) of ciprofloxacin and delafloxacin were determined using the broth microdilution method according to EUCAST recommendations. In addition to standard MHB (pH 7.3 ± 0.1), assays were performed in MHB adjusted to pH 6.0 ± 0.1 and 5.0 ± 0.1 by adding HCl (Sigma-Aldrich, Madrid, Spain) before sterilization (5). These pH values were chosen because approximately 90% of urine samples display pH <7 and 40% display pH <6 during a UTI caused by *E. coli* (5). The final pH values were determined before use. MIC determinations were performed in triplicate for each strain and pH condition.

### Time-killing assays

Time-killing assays (TKAs) were performed using the WT *E. coli* ATCC 25922, EC02 strain carrying the common chromosomal mutation (*gyrA*-S83L), and EC11 strain carrying the most prevalent PMQR determinant (*qnrB1*). Overnight cultures were grown in quinolone-free MHB at 37°C. Bacterial suspensions were adjusted to 5 × 10⁵ CFU/mL and inoculated into fresh MHB at pH 7.3, or 5.0, supplemented with ciprofloxacin or delafloxacin (pH was controlled after addition of antibiotic and remained stable). Seven antibiotic concentrations, ranging from 0.125-fold to 8-fold the MIC for each strain (in log₂ increments), were tested. Antibiotic-free media served as controls. Viable counts were obtained by plating serial dilutions onto MHA plates and incubating at 37°C for up to 24 h. The lower limit of quantification was 10² CFU/mL. Each experiment was performed in duplicate.

### Mechanism-based modeling

A semi-mechanistic-based mathematical model was developed using Monolix version 2024R1 (Lixoft, Antony, France) to quantitatively describe bacterial killing and regrowth (supplementary materials). Model parameters were estimated using the stochastic approximation expectation maximization algorithm, with standard errors calculated through linearization of the Fisher information matrix. The model incorporated two distinct initial bacterial subpopulations: susceptible (S) and pre-existing resistant (R) bacteria. The initial resistant population was fixed to 1 CFU due to the low spontaneous mutation frequency (≤10⁻⁸) (12). The total viable population (Total_pop_) was described as follows:


$$Total_{pop}=S+R$$

At time zero, *S*_*₀*_ was estimated as a model parameter (Initial_popS_) and *R*_*₀*_ was fixed to 1. The growth inhibition of susceptible population by quinolones (inhiB_*S*_) was described using an *E*_max_ model:


$${inhiB}_{S}=\frac{E_{max}\times C_{\text{drug}}{}^{h}}{{EC50}_{S}{}^{h}+C_{\text{drug}}{}^{h}}$$

where *E*_max_ is the maximum drug effect constant, EC50_*S*_ represents the drug concentration producing 50% of *E*_max_, *C*_drug_ represents the free drug concentration, and *h* represents the Hill coefficient. Drug concentrations were modeled using a one-compartment model with a first-order elimination rate constant (*k*_*e*_). Given the high chemical stability of quinolones (98.8% remaining after 24 h), *k*_*e*_ for TKA was fixed to 0.0005 h⁻¹ (13). Protein binding was taken into account with *C*_drug_ calculated as follows:


$$C_{drug}=C_{c}\times Ff_{prot}$$

where *C*_*c*_ is the total drug concentration and Ff_prot_ is the fraction of free drug. For TKA, the Ff_prot_ was fixed to 1. The concentration of susceptible bacteria was described as follows:


$$\frac{dS}{dt}=r_{S}\times \left(1-\frac{S+R}{Pop_{max}}\right)\times S-{inhiB}_{S}\times S$$

where *r*_*S*_ is the specific growth rate constant and Pop_max_ is the maximal population. The resistant bacterial subpopulation grew similarly, with a reduced growth rate (*r*_*R*_) estimated as a fraction (Fr) of *r*_*S*_ (bounded between 0 and 1):


$$r_{R}=r_{S}\times Fr$$

Monte Carlo simulations (*n* = 10,000) were conducted using free drug concentration time profiles for simulated dosing regimens: ciprofloxacin 400 mg q12h IV and delafloxacin 350 mg q12h IV. Pharmacokinetic parameters are provided in the supplementary material (Table S2).

### Statistical analysis

Statistical analyses were performed using R software (version 4.3.0) and Monolix 2024R1 (Lixoft, Antony, France). Descriptive statistics are presented as median with interquartile range (IQR) for continuous variables. Paired comparisons between ciprofloxacin and delafloxacin MICs at each pH condition were performed using the Wilcoxon signed-rank test for paired samples. Comparisons of susceptibility rates between WT and PMQR-carrying strains were assessed using McNemar’s test for paired categorical data. For pharmacodynamic parameter comparisons, EC_50_ values were compared using Wald tests on log-transformed parameters, accounting for parameter uncertainty through relative standard errors. Statistical comparisons of EC_50_ ratios between conditions were performed using bootstrap confidence intervals (95% CI) derived from Monte Carlo simulations (*n* = 10,000). Significant differences in EC_50_ values were defined as non-overlapping 95% CI and *P* < 0.05. Statistical significance was set at *P* < 0.05 for all analyses.

## RESULTS

### Antibiotic susceptibility testing

**Fig 1:**
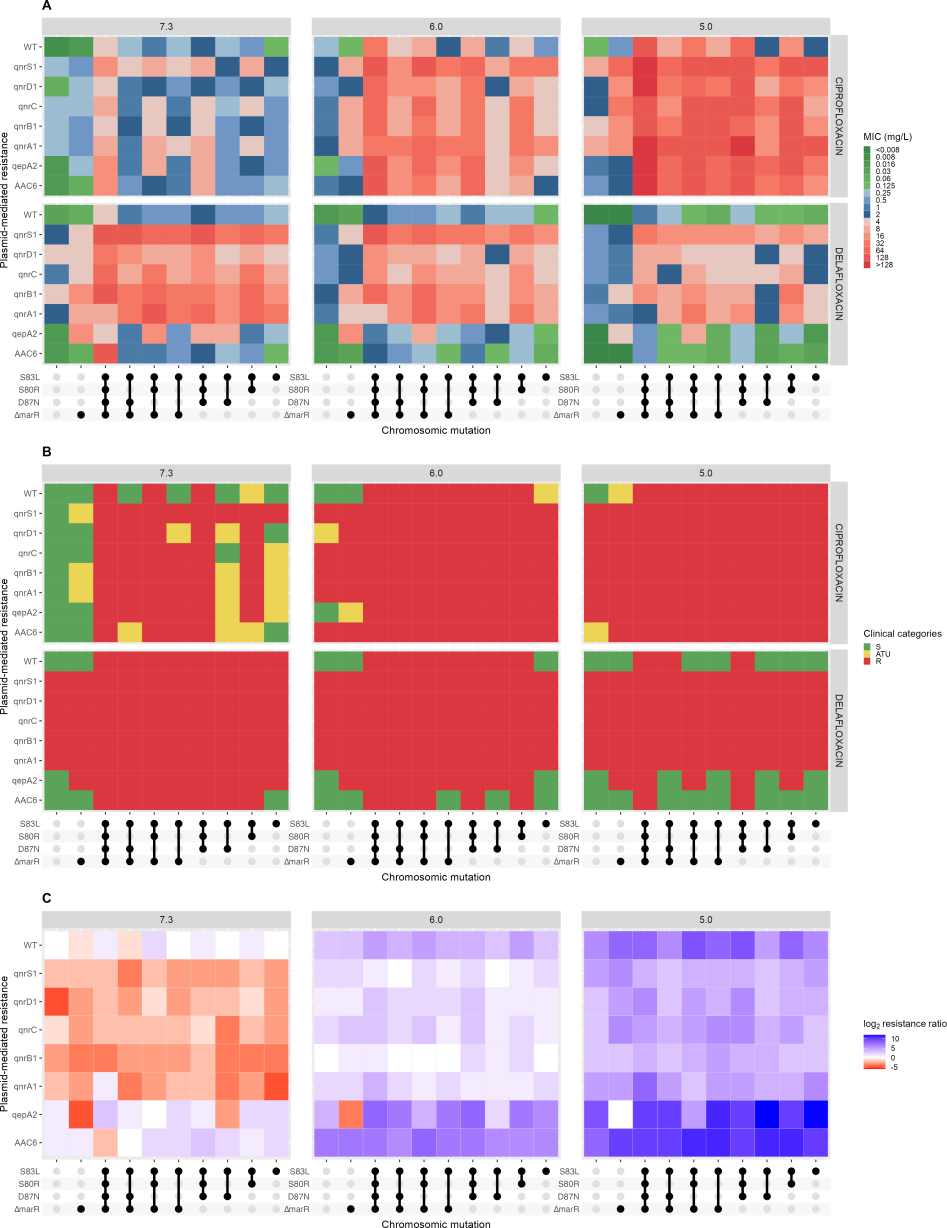
Impact of pH on bacteriostatic activity of ciprofloxacin and delafloxacin against *E. coli* strains with different resistance mechanisms. (**A**) MIC distribution across different chromosomic mutations and plasmid-mediated resistance mechanisms at pH 5.0, 6.0, and 7.3 for ciprofloxacin and delafloxacin. (**B**) Clinical categories (susceptible, intermediate, resistant) based on EUCAST breakpoints for the same resistance mechanisms and conditions. (**C**) Resistance ratio calculated as log2 of the ratio: (MIC_ciprofloxacin_mutant/MIC_ciprofloxacin_wild-type_pH7.3)/(MIC_delafloxacin_mutant/MIC_delafloxacin_wild-type_pH7.3), with red indicating preferential resistance to delafloxacin and blue indicating preferential resistance to ciprofloxacin. Mutations: S83L and S80R in gyrA, D87N in gyrB, and ∆marR (marR deletion). Plasmid-mediated resistance genes tested include qnr variants and aac(6')-Ib-cr. S: susceptible; ATU: area of technical uncertainty; R: resistant.

Overall, ciprofloxacin MICs increased with decreasing pH, rising from an MIC₉₀ value of 8 mg/L at pH 7.3 to 128 mg/L at pH 5.0 (Fig. 1A; Table S3). Conversely, delafloxacin demonstrated enhanced activity under acidic conditions, with MIC₉₀ values decreasing from 64 mg/L at pH 7.3 to 16 mg/L at pH 5.0. At physiological pH, ciprofloxacin showed superior activity with a median (IQR) MIC of 1 (0.25–4) mg/L compared to delafloxacin at 8 (1–32) mg/L (*P* < 0.001, Wilcoxon test). However, this relationship reversed at acidic pH levels, where delafloxacin exhibited significantly lower MICs: 4 (0.5–16) mg/L versus 8 (4–32) mg/L for ciprofloxacin at pH 6.0 (*P* < 0.001, Wilcoxon test) and 2 (0.125–8) mg/L versus 64 (16–128) mg/L at pH 5.0 (*P* < 0.001, Wilcoxon test). Analysis of isogenic strain pairs revealed distinct differential impacts of PMQR mechanisms on delafloxacin activity. Fold changes were calculated as the ratio of MIC values (MIC with PMQR/MIC without PMQR) for each strain pair sharing identical chromosomal mutations. Both *qepA2* and *aac(6')-Ib-cr* conferred minimal additional resistance to delafloxacin, with median fold changes of 1 (meaning no additional resistance) across all pH conditions tested. In contrast, *qnr* gene variants substantially compromised delafloxacin efficacy, generating median fold changes of 64 (IQR: 16–128) at pH 7.3 and 67 (IQR: 32–128) at pH 5.0.

According to EUCAST breakpoints (susceptibility defined as MIC ≤0.125 mg/L for delafloxacin and ≤0.25 mg/L for ciprofloxacin), the susceptibility patterns differed markedly between antibiotics and pH conditions (Fig. 1B). At physiological pH, 25.9% (21/81) of strains were susceptible to ciprofloxacin compared to only 8.6% (7/81) for delafloxacin (*P* < 0.001, McNemar’s test). This relationship reversed under acidic conditions: at pH 5.0, susceptibility to ciprofloxacin dropped to 2.5% (2/81), while delafloxacin susceptibility increased to 25.9% (21/81) (*P* < 0.001, McNemar’s test). Among PMQR-negative strains, only 3.7% remained resistant to delafloxacin at pH 5.0 (mutation profiles: S83L-S80R-D87N-Δ*marR*, S83L-D87N-Δ*marR*, S83L-S80R-D87N). Among PMQR mechanisms, *qnr* genes led to 0% delafloxacin susceptibility across all conditions (0/150 strain-pH combinations), while *qepA2* (8/30 strain-pH combinations, 26.7%, *P* = 0.22) and *aac(6')-Ib-cr* (16/30 strain-pH combinations, 53.3%, *P* = 0.13, McNemar’s test) showed comparable susceptibility frequencies to isogenic strains not carrying PMQR (15/33 strain-pH combinations, 45.5%). Ciprofloxacin susceptibility at pH 5.0 was restricted to WT isolates only.

The differential impact of resistance mechanisms on ciprofloxacin versus delafloxacin revealed distinct patterns across pH conditions (Fig. 1C). At physiological pH (7.3), most resistance mechanisms preferentially impacted delafloxacin, with 67.5% of strain-mechanism combinations showing negative log₂ ratios (median ratio: −2.0, range: −5.0 to 3.0). This differential impact was most pronounced for *qnr* genes, which consistently showed strong preferential resistance to delafloxacin across all chromosomal backgrounds (median log₂ ratio: −3.0). In contrast, *qepA2* and *aac(6')-Ib-cr* preferentially affected ciprofloxacin (median log₂ ratios: 1.0 and 1.5, respectively). The differential resistance pattern shifted notably with decreasing pH, with 98.8% of resistance mechanisms showing preferential impact on ciprofloxacin at pH 5.0.

### Time-killing assays

**Fig 2:**
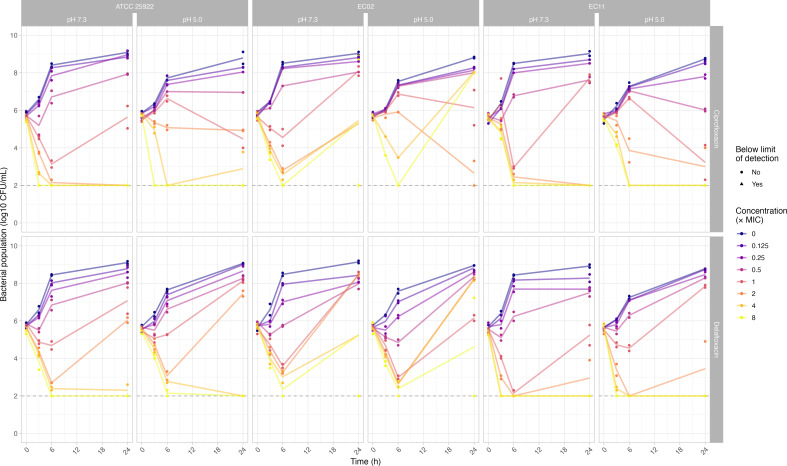
Time-kill assays of ciprofloxacin and delafloxacin against three *E. coli* strains at pH 7.3 and 5.0. Individual data points represent colony-forming unit (CFU) counts from independent experiments, with filled circles indicating values above the limit of detection and triangles indicating values below the limit of detection. Solid lines represent mean values for each concentration. The horizontal dashed line indicates the limit of detection (2 log₁₀ CFU/mL). Color coding represents different antibiotic concentrations relative to each strain’s MIC (0× to 8× MIC).

TKA results are displayed in Fig. 2. Against WT *E. coli* ATCC 25922, bactericidal activity at 24 h was observed for both ciprofloxacin and delafloxacin at eightfold MIC concentrations, and these bactericidal activities were not affected by the acidic environment. However, the acidic environment impaired ciprofloxacin activity against *E. coli* ECO2 (*gyrA*-S83L), showing regrowth at 24 h for all tested concentrations, while delafloxacin demonstrated bactericidal activity at eightfold MIC in one replicate under acidic conditions. Against *E. coli* EC11 (*qnrB1*), ciprofloxacin showed bactericidal activity at twofold MIC at pH 7.3 and required fourfold MIC at pH 5.0, whereas delafloxacin required eightfold MIC at neutral pH but only fourfold MIC in the acidic environment.

### Mechanism-based modeling

Observations from TKA were confirmed by the semi-mechanistic-based modeling (Table S2). Pharmacodynamic parameter comparisons on log-transformed EC_50_ values showed statistically significant differences between antibiotics at all pH conditions (*P* < 0.001, Wald test). The EC_50_ ratios (ciprofloxacin/delafloxacin) increased dramatically from physiological pH 7.3 to acidic pH 5.0, reaching 33-fold for the WT strain (95% CI: 21.0–52.9) and 92-fold for the GyrA mutant (95% CI: 87.8–97.3). Conversely, ciprofloxacin activity decreased 6- to 20-fold at acidic pH, while delafloxacin activity improved 2- to 3-fold under the same conditions across all tested strains.

**Fig 3:**
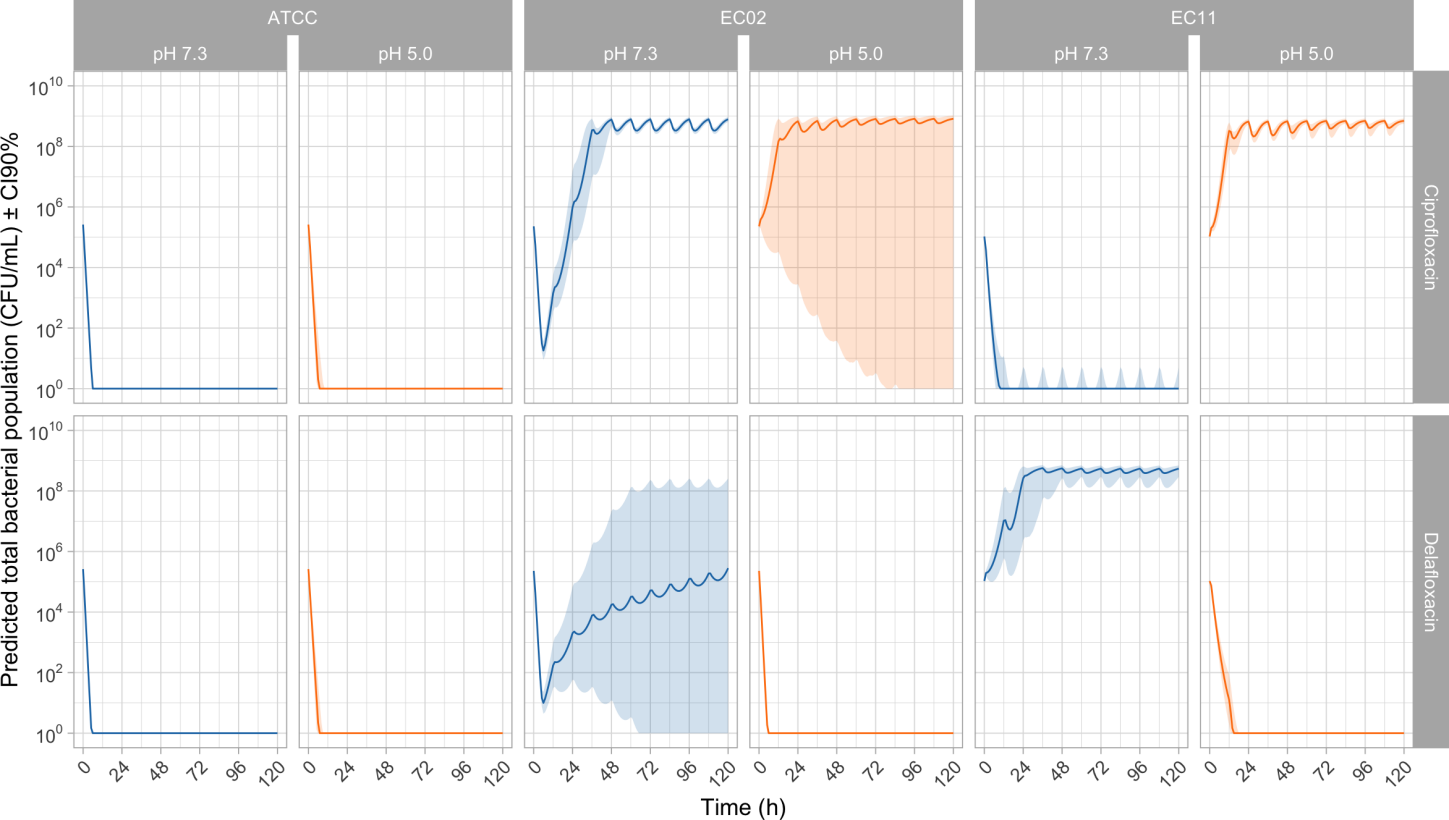
Predicted bacterial population dynamics under ciprofloxacin and delafloxacin exposure at pH 7.3 and 5.0. Predicted total bacterial populations (CFU/mL ± 90% CI) following simulated clinical dosing of ciprofloxacin (400 mg q12h IV) and delafloxacin (350 mg q12h IV) against three *E. coli* strains under physiological (pH 7.3, blue lines) and acidic (pH 5.0, orange lines) conditions over 120 h. ATCC 25922 (WT strain), EC02 (*gyrA*-S83L chromosomal mutant), and EC11 (*qnrB1* PMQR-carrying strain). Solid lines represent median predictions from the semi-mechanistic pharmacokinetic-pharmacodynamic model, while shaded areas indicate 90% CI derived from Monte Carlo simulations (*n* = 10,000).

Simulated bacterial populations after simulated exposure to both drugs (ciprofloxacin: 400 mg q12h IV, delafloxacin: 350 mg q12h IV) demonstrated pH- and strain-dependent patterns (Fig. 3). Against WT *E. coli* ATCC 25922, both drugs achieved sustained bacterial killing without regrowth, irrespective of pH conditions. Against the chromosomal mutant EC02 (*gyrA*-S83L), ciprofloxacin’s bactericidal effect was abolished at acidic pH with extensive bacterial regrowth, while delafloxacin maintained effective suppression at both pH conditions. Notably, delafloxacin’s activity against EC02 at physiological pH exhibited substantial variability, indicating borderline efficacy that improved significantly under acidic conditions. Uncertainty, displayed by a wide 90% CI, was observed for simulated bacterial populations of EC02 after exposure to ciprofloxacin in acidic environment. Against the PMQR-producing strain EC11 (*qnrB1*), acidic pH resulted in a change regarding outcome. For ciprofloxacin, at neutral pH, a total bacterial suppression was simulated, while at pH 5.0, early bacterial regrowth (at h = 12) was simulated. For delafloxacin, at neutral pH, an early therapeutic failure was simulated with bacterial regrowth at t = 24 h, and in acidic environment, a total bacterial suppression with bactericidal activity before t = 24 h was simulated with low uncertainty.

## DISCUSSION

This study provides a comprehensive evaluation of pH effects on fluoroquinolone activity against a systematically characterized collection of *E. coli* strains harboring diverse resistance mechanisms. Our principal findings demonstrate an important reversal in the relative activities of ciprofloxacin and delafloxacin as pH decreases from physiological to acidic conditions, with important implications for clinical practice and antimicrobial stewardship.

Our results confirm and extend previous observations regarding the pH-dependent activity of fluoroquinolones. The decreased activity of ciprofloxacin under acidic conditions aligns with earlier reports (5). The enhanced activity of delafloxacin under acidic conditions represents a unique pharmacological property that distinguishes it from other fluoroquinolones. While previous studies have reported maintained or improved delafloxacin activity at low pH, our data quantify this effect across multiple resistance backgrounds using both bacteriostatic and bactericidal methodologies. The 2- to 3-fold improvement in delafloxacin activity at pH 5.0 compared to physiological pH contrasts sharply with the 6- to 20-fold decrease observed for ciprofloxacin, resulting in EC_50_ ratios (ciprofloxacin/delafloxacin) exceeding 90-fold for certain resistance profiles. These differential pH effects can be explained by distinct uptake mechanisms. Impaired uptake of zwitterionic fluoroquinolones occurs in acidic environments, while uptake is enhanced for delafloxacin due to its unique chemical properties (4, 7). Indeed, delafloxacin possesses unique chemical properties with an anionic character at neutral pH that shifts to a predominantly neutral form at acidic pH. This pH-dependent structural change allows delafloxacin at acidic pH to cross bacterial membranes more efficiently through passive diffusion, leading to higher intracellular concentrations (6). This mechanism is supported by our observation that delafloxacin activity improvement under acidic conditions is less pronounced in strains with efflux pump overexpression (*marR* mutants), suggesting that efflux-mediated resistance can partially overcome the pH-related uptake advantage. These findings align with a recent study by Bösch et al. that examined resistance evolution in eight *E. coli* clinical strains exposed to ciprofloxacin and delafloxacin at different pH values (14). The authors demonstrated that delafloxacin resistance evolution was significantly slower at pH 6.0 than at pH 7.3, with mutations in efflux-related genes (*emrR*, *marR*) playing a prominent role in delafloxacin resistance development.

The resistance to delafloxacin conferred by *qnr* genes across all pH conditions indicates that target protection mechanisms can override the pH-dependent uptake advantages. This finding suggests that while pH affects drug-target interaction kinetics, the physical shielding of DNA gyrase and topoisomerase IV by Qnr proteins provides protection regardless of intracellular drug concentration. These results are consistent with previous studies demonstrating *qnr* gene impact on delafloxacin resistance among *E. coli* clinical strains (15). Conversely, *qepA2* and *aac(6')-Ib-cr* demonstrated minimal impact on delafloxacin activity. The *aac(6')-Ib-c* enzyme acetylates quinolones with a piperazinyl substituent that is absent in delafloxacin (6, 16). QepA2 belongs to the MFS efflux pump family specific to hydrophilic quinolones; the amphiphilic character of delafloxacin may explain the minimal impact of *qepA2* on delafloxacin MIC (17). To note, delafloxacin maintained clinical susceptibility even for combinations of up to four resistance mechanisms in which no *qnr* genes were included (Fig. 1B). These differences may be important at the therapeutic level due to the higher prevalence of chromosomal (compared to plasmid-mediated) mechanisms of quinolone resistance in clinical isolates (18, 19).

Our results may have several important clinical implications for delafloxacin use in practice. Given that simulated clinical regimens demonstrated substantial differences in microbiological outcomes between acidic and neutral pH conditions, current AST may inadequately predict delafloxacin efficacy at infection sites. Specifically, standard AST methods performed at physiological pH could underestimate delafloxacin potency in acidic microenvironments, such as abscesses, biofilms, or urinary tract infections. This suggests that AST protocols should evolve to incorporate microenvironmental characteristics that significantly modify drug activity (1, 2). Nevertheless, these laboratory-based observations require validation through dynamic *in vitro* models and ultimately *in vivo* studies to confirm their clinical relevance.

The major strengths of this study include the use of isogenic strains eliminating confounding genetic backgrounds, comprehensive evaluation of major resistance mechanisms, and integration of static and dynamic antimicrobial testing with semi-mechanism-based mathematical modeling.

Several limitations should be acknowledged. First, laboratory media may not fully represent the complex chemical environment of infection sites containing proteins, ions, and other factors influencing drug activity. Second, pH values were not monitored during experiments, and we cannot exclude pH fluctuations due to bacterial metabolism that may have influenced antibiotic activity. Additionally, the study examined only *E. coli*, and results may not be generalizable to other Gram-negative pathogens causing infections in acidic environments. Also, semi-mechanistic models may not fully represent the dynamic bacterial responses to antimicrobial exposure, which can limit their predictive accuracy, particularly under conditions outside the range of the experimental data used for model fitting.

Several important research directions emerge from this work. Development of pH-adjusted susceptibility testing methods could improve prediction of clinical outcomes for infections in acidic sites. Clinical studies correlating infection site pH measurements with treatment outcomes could validate the clinical relevance of our *in vitro* findings, particularly for infections in anatomical sites with variable pH.

### Conclusions

This study demonstrates that environmental pH profoundly influences the relative activities of ciprofloxacin and delafloxacin against *E. coli*, with implications extending beyond simple MIC comparisons to encompass complex interactions with resistance mechanisms. The resistance conferred by *qnr* genes regardless of pH emphasizes the continued importance of resistance mechanism characterization for optimal therapeutic decision-making. These findings support the need for infection site-specific antibiotic selection strategies and highlight the potential value of incorporating environmental factors into AST protocols.

## SUPPLEMENTAL MATERIAL

Text S1 spectrum.02338-25-s0001.txt Structural model used in Monolix for PK/PD modeling and simulation.

Table S1 spectrum.02338-25-s0002.docx Genotypes of isogenic strains used.

Table S2 spectrum.02338-25-s0003.docx Parameter estimates of the pharmacodynamic model for the three strains according to drug and pH.

Table S3 spectrum.02338-25-s0004.docx Modal MICs of ciprofloxacin and delafloxacin according to pH against all strains.

ASM does not own the copyrights to Supplemental Material that may be linked to, or accessed through, an article. The authors have granted ASM a non-exclusive, world-wide license to publish the Supplemental Material files. Please contact the corresponding author directly for reuse.
