# Supplementary material for: Effect of pH on antimicrobial activity of delafloxacin against Escherichia coli isogenic strains carrying diverse chromosomal and plasmid-mediated fluoroquinolone resistance mechanisms
Source: Microbiol Spectr. 2025 Oct 30;13(12):e02338-25. doi: 10.1128/spectrum.02338-25 (PMC12671115; doi:10.1128/spectrum.02338-25)
Supplement: Table S1 — Genotypes of isogenic strains used. [file spectrum.02338-25-s0002.docx]

| **Strain^a^** | ***gyrA1*** | ***gyrA2*** | ***parC*** | ***marR*** | **PMQR** | **Source or reference** |
| --- | --- | --- | --- | --- | --- | --- |
| ATCC 25922 | - | - | - | - | - |  |
| ATCC 25922 pBK-CMV | - | - | - | - | - |  |
| EC01 | - | - | - | Δ | - | [1] |
| EC02 | S83L | - | - | - | - | [2] |
| EC03 | S83L | - | - | Δ | - | [1] |
| EC04 | S83L | - | S80R | - | - | [2] |
| EC05 | S83L | - | S80R | Δ | - | [1] |
| EC06 | S83L | D87N | - | - | - | [1] |
| EC07 | S83L | D87N | - | Δ | - | [1] |
| EC08 | S83L | D87N | S80R | - | - | [1] |
| EC09 | S83L | D87N | S80R | Δ | - | [1] |
| EC10 | - | - | - | - | pBK-qnrA1 | [2] |
| EC11 | - | - | - | - | pBK-qnrB1 | [2] |
| EC12 | - | - | - | - | pBK-qnrC | [1] |
| EC13 | - | - | - | - | pBK-qnrD1 | [1] |
| EC14 | - | - | - | - | pBK-qnrS1 | [2] |
| EC15 | - | - | - | Δ | pBK-qnrA1 | [1] |
| EC16 | - | - | - | Δ | pBK-qnrB1 | [1] |
| EC17 | - | - | - | Δ | pBK-qnrC | [1] |
| EC18 | - | - | - | Δ | pBK-qnrD1 | [1] |
| EC19 | - | - | - | Δ | pBK-qnrS1 | [1] |
| EC20 | S83L | - | - | - | pBK-qnrA1 | [2] |
| EC21 | S83L | - | - | - | pBK-qnrB1 | [2] |
| EC22 | S83L | - | - | - | pBK-qnrC | [1] |
| EC23 | S83L | - | - | - | pBK-qnrD1 | [1] |
| EC24 | S83L | - | - | - | pBK-qnrS1 | [2] |
| EC25 | S83L | - | - | Δ | pBK-qnrA1 | [1] |
| EC26 | S83L | - | - | Δ | pBK-qnrB1 | [1] |
| EC27 | S83L | - | - | Δ | pBK-qnrC | [1] |
| EC28 | S83L | - | - | Δ | pBK-qnrD1 | [1] |
| EC29 | S83L | - | - | Δ | pBK-qnrS1 | [1] |
| EC30 | S83L | - | S80R | - | pBK-qnrA1 | [2] |
| EC31 | S83L | - | S80R | - | pBK-qnrB1 | [2] |
| EC32 | S83L | - | S80R | - | pBK-qnrC | [1] |
| EC33 | S83L | - | S80R | - | pBK-qnrD1 | [1] |
| EC34 | S83L | - | S80R | - | pBK-qnrS1 | [2] |
| EC35 | S83L | - | S80R | Δ | pBK-qnrA1 | [1] |
| EC36 | S83L | - | S80R | Δ | pBK-qnrB1 | [1] |
| EC37 | S83L | - | S80R | Δ | pBK-qnrC | [1] |
| EC38 | S83L | - | S80R | Δ | pBK-qnrD1 | [1] |
| EC39 | S83L | - | S80R | Δ | pBK-qnrS1 | [1] |
| EC40 | S83L | D87N | - | - | pBK-qnrA1 | [1] |
| EC41 | S83L | D87N | - | - | pBK-qnrB1 | [1] |
| EC42 | S83L | D87N | - | - | pBK-qnrC | [1] |
| EC43 | S83L | D87N | - | - | pBK-qnrD1 | [1] |
| EC44 | S83L | D87N | - | - | pBK-qnrS1 | [1] |
| EC45 | S83L | D87N | - | Δ | pBK-qnrA1 | [1] |
| EC46 | S83L | D87N | - | Δ | pBK-qnrB1 | [1] |
| EC47 | S83L | D87N | - | Δ | pBK-qnrC | [1] |
| EC48 | S83L | D87N | - | Δ | pBK-qnrD1 | [1] |
| EC49 | S83L | D87N | - | Δ | pBK-qnrS1 | [1] |
| EC50 | S83L | D87N | S80R | - | pBK-qnrA1 | [1] |
| EC51 | S83L | D87N | S80R | - | pBK-qnrB1 | [1] |
| EC52 | S83L | D87N | S80R | - | pBK-qnrC | [1] |
| EC53 | S83L | D87N | S80R | - | pBK-qnrD1 | [1] |
| EC54 | S83L | D87N | S80R | - | pBK-qnrS1 | [1] |
| EC55 | S83L | D87N | S80R | Δ | pBK-qnrA1 | [1] |
| EC56 | S83L | D87N | S80R | Δ | pBK-qnrB1 | [1] |
| EC57 | S83L | D87N | S80R | Δ | pBK-qnrC | [1] |
| EC58 | S83L | D87N | S80R | Δ | pBK-qnrD1 | [1] |
| EC59 | S83L | D87N | S80R | Δ | pBK-qnrS1 | [1] |
| EC60 | - | - | - | - | pBK-qepA2 | [3] |
| EC61 | - | - | - | Δ | pBK-qepA2 | [3] |
| EC62 | S83L | - | - | - | pBK-qepA2 | [3] |
| EC63 | S83L | - | - | Δ | pBK-qepA2 | [3] |
| EC64 | S83L | - | S80R | - | pBK-qepA2 | [3] |
| EC65 | S83L | - | S80R | Δ | pBK-qepA2 | [3] |
| EC66 | S83L | D87N | - | - | pBK-qepA2 | [3] |
| EC67 | S83L | D87N | - | Δ | pBK-qepA2 | [3] |
| EC68 | S83L | D87N | S80R | - | pBK-qepA2 | [3] |
| EC69 | S83L | D87N | S80R | Δ | pBK-qepA2 | [3] |
| EC70 | - | - | - | - | pBK-aac(6’)-Ib-cr | [4] |
| EC71 | - | - | - | Δ | pBK-aac(6’)-Ib-cr | [4] |
| EC72 | S83L | - | - | - | pBK-aac(6’)-Ib-cr | [4] |
| EC73 | S83L | - | - | Δ | pBK-aac(6’)-Ib-cr | [4] |
| EC74 | S83L | - | S80R | - | pBK-aac(6’)-Ib-cr | [4] |
| EC75 | S83L | - | S80R | Δ | pBK-aac(6’)-Ib-cr | [4] |
| EC76 | S83L | D87N | - | - | pBK-aac(6’)-Ib-cr | [4] |
| EC77 | S83L | D87N | - | Δ | pBK-aac(6’)-Ib-cr | [4] |
| EC78 | S83L | D87N | S80R | - | pBK-aac(6’)-Ib-cr | [4] |
| EC79 | S83L | D87N | S80R | Δ | pBK-aac(6’)-Ib-cr | [4] |

**Table S1:** Genotypes of isogenic strains

^a^Genotype. Strains are isogenic to *E. coli* ATCC 25922 and carry only the chromosomal modifications and/or the PMQR gene shown

PMQR= plasmid-mediated quinolone resistance

[1] Machuca J, Briales A, Labrador G, Díaz-de-Alba P, López-Rojas R, Docobo-Pérez F, et al. Interplay between plasmid-mediated and chromosomal-mediated fluoroquinolone resistance and bacterial fitness in Escherichia coli. J Antimicrob Chemother. 2014 Dec 1;69(12):3203–15.

[2] Briales A, Rodríguez-Martínez JM, Velasco C, Díaz De Alba P, Domínguez-Herrera J, Pachón J, et al. *In Vitro* Effect of *qnrA1* , *qnrB1* , and *qnrS1* Genes on Fluoroquinolone Activity against Isogenic *Escherichia coli* Isolates with Mutations in *gyrA* and *parC*. Antimicrob Agents Chemother. 2011 Mar;55(3):1266–9.

[3] Machuca J, Ortiz M, Recacha E, Díaz-De-Alba P, Docobo-Perez F, Rodríguez-Martínez JM, et al. Impact of AAC(6′)-Ib-cr in combination with chromosomal-mediated mechanisms on clinical quinolone resistance in *Escherichia coli*. J Antimicrob Chemother. 2016 Nov;71(11):3066–71.

[4] Machuca J, Briales A, Díaz-de-Alba P, Martínez-Martínez L, Pascual Á, Rodríguez-Martínez JM. Effect of the efflux pump QepA2 combined with chromosomally mediated mechanisms on quinolone resistance and bacterial fitness in Escherichia coli. J Antimicrob Chemother. 2015 Sep;70(9):2524–7.
