# Supplementary material for: Effect of pH on antimicrobial activity of delafloxacin against Escherichia coli isogenic strains carrying diverse chromosomal and plasmid-mediated fluoroquinolone resistance mechanisms
Source: Microbiol Spectr. 2025 Oct 30;13(12):e02338-25. doi: 10.1128/spectrum.02338-25 (PMC12671115; doi:10.1128/spectrum.02338-25)
Supplement: Table S2 — Parameter estimates of the pharmacodynamic model for the three strains according to drug and pH. [file spectrum.02338-25-s0003.docx]

|  |  | **ATCC25922** | | | | **EC02** | | | | **EC11** | | | |
| --- | --- | --- | --- | --- | --- | --- | --- | --- | --- | --- | --- | --- | --- |
|  |  | CIP | | DEL | | CIP | | DEL | | CIP | | DEL | |
|  |  | pH 7.3 | pH 5.0 | pH 7.3 | pH 5.0 | pH 7.3 | pH 5.0 | pH 7.3 | pH 5.0 | pH 7.3 | pH 5.0 | pH 7.3 | pH 5.0 |
| **Bacterial parameters** | |  |  |  |  |  |  |  |  |  |  |  |  |
|  | Initial S population, CFU/mL (%R.S.E) | 2.6e5 (2.1) | | | | 2.29e5 (2.6) | | | | 1.06e5 (1.4) | | | |
|  | Growth rate S population, h-1 (%R.S.E) | 1.03 (5.5) | | | | 1.03 (7.1) | | | | 1.09 (8.7) | | | |
|  | Maximum S population, CFU/mL (%R.S.E) | 1.03e9(17.6) | | | | 9.67e8 (10.8) | | | | 7.35e8 (11.2) | | | |
|  | Relative growth rate of R compared to S (%R.S.E.) | 0.95 (0.16) | | | | 0.95 (0.15) | | | | 0.97 (0.20) | | | |
| **Drug parameters^a^** | | | | | | | | | | | | | |
|  | Volume of distribution (L) | 140 | | 34.2 | | 140 | | 34.2 | | 140 | | 34.2 | |
|  | Constant of elimination (h-1) | 0.165 | | 0.064 | | 0.165 | | 0.064 | | 0.165 | | 0.064 | |
|  | Free fraction of drug | 0.75 | | 0.16 | | 0.75 | | 0.16 | | 0.75 | | 0.16 | |
| **Drug activity parameters** | |  |  |  |  |  |  |  |  |  |  |  |  |
|  | Maximum Effect, h-1 (%R.S.E.) | 3.22 (1.6) | 3.47 (13.6) | 3.5 (0.1) | 2.99 (3.3) | 3.48 (0.7) | 3.47 (8.8) | 3.44 (0.7) | 3.49 (0.1) | 3.46 (5.8) | 3.46 (9.93) | 3.5 (12.6) | 3.49 (0.1) |
|  | Hill coefficient (% R.S.E.) | 1.81 (1.2) | 1.1 (14.3) | 1.06 (1.8) | 1.33 (1.5) | 1.28 (1.8) | 1 (38.6) | 1.07 (5.2) | 1.31 (0.1) | 1.09 (0.1) | 1.87 (2.66) | 1.58 (3.4) | 1.01 (13.8) |
|  | EC50 on S population, mg/L (% R.S.E.) | 0.0084 (11.7) | 0.17 (20.8) | 0.016 (2.8) | 0.0051 (11.0) | 0.35 (2.83) | 5.73 (2.6) | 0.14 (8.0) | 0.062 (0.1) | 0.48 (7.4) | 3.05 (4.26) | 2.66 (5.1) | 0.95 (2.1) |
|  | EC50 on R population, mg/L (%R.S.E.) | 0.014 (2.4) | 0.45 (45.2) | 0.12 (8.5) | 0.038 (27.3) | 4.81 (2.31) | 14.4 (25.4) | 5.24 (7.2) | 1.45 (0.1) | 1.63 (5.8) | 4.7 (15.7) | 8.08 (23.9) | 2.34 (2.7) |

**Table S2:** Parameters estimates of the pharmacodynamic model for the three strains according to drug and pH

^a^ drug parameters were adapted from Shiu J, Ting G, Kiang TK. Clinical Pharmacokinetics and Pharmacodynamics of Delafloxacin. Eur J Drug Metab Pharmacokinet. 2019 Jun 1;44(3):305–17. and Catchpole C, Andrews JM, Woodcock J, Wise R. The comparative pharmacokinetics and tissue penetration of single-dose ciprofloxacin 400 mg iv and 750 mg po. J Antimicrob Chemother. 1994;33(1):103–10.
