# Supplementary material for: Effect of pH on antimicrobial activity of delafloxacin against Escherichia coli isogenic strains carrying diverse chromosomal and plasmid-mediated fluoroquinolone resistance mechanisms
Source: Microbiol Spectr. 2025 Oct 30;13(12):e02338-25. doi: 10.1128/spectrum.02338-25 (PMC12671115; doi:10.1128/spectrum.02338-25)
Supplement: Table S3 — Modal MICs of ciprofloxacin and delafloxacin according to pH against all strains. [file spectrum.02338-25-s0004.docx]

| Strains | Ciprofloxacin MIC (mg/L) | | | Delafloxacin MIC (mg/L) | | |
| --- | --- | --- | --- | --- | --- | --- |
|  | pH 7.3 | pH 6.0 | pH 5.0 | pH 7.3 | pH 6.0 | pH 5.0 |
| ATCC_25922 | 0.008 | 0.03 | 0.125 | 0.016 | 0.008 | 0.004 |
| ATCC_25922_pBK-CMV | 0.004 | 0.25 | 0.125 | 0.03 | 0.016 | 0.004 |
| EC01 | 0.016 | 0.125 | 0.5 | 0.06 | 0.03 | 0.004 |
| EC02 | 0.125 | 0.5 | 2 | 0.25 | 0.125 | 0.06 |
| EC03 | 0.25 | 2 | 8 | 0.5 | 0.25 | 0.06 |
| EC04 | 0.5 | 4 | 16 | 0.5 | 0.25 | 0.125 |
| EC05 | 1 | 8 | 32 | 0.5 | 0.5 | 0.125 |
| EC06 | 0.25 | 1 | 2 | 0.5 | 0.25 | 0.125 |
| EC07 | 0.25 | 4 | 8 | 1 | 0.5 | 0.25 |
| EC08 | 2 | 8 | 64 | 2 | 1 | 0.25 |
| EC09 | 4 | 32 | 128 | 4 | 2 | 1 |
| EC10 | 0.25 | 2 | 8 | 2 | 1 | 0.5 |
| EC11 | 0.25 | 1 | 4 | 4 | 2 | 1 |
| EC12 | 0.25 | 1 | 2 | 1 | 0.5 | 0.5 |
| EC13 | 0.06 | 0.5 | 2 | 4 | 0.5 | 0.5 |
| EC14 | 0.25 | 2 | 8 | 2 | 1 | 0.5 |
| EC15 | 0.5 | 8 | 16 | 8 | 4 | 1 |
| EC16 | 0.5 | 8 | 16 | 16 | 8 | 4 |
| EC17 | 0.25 | 8 | 32 | 4 | 2 | 1 |
| EC18 | 0.25 | 2 | 16 | 4 | 2 | 1 |
| EC19 | 0.5 | 8 | 64 | 4 | 4 | 2 |
| EC20 | 0.5 | 16 | 128 | 32 | 8 | 4 |
| EC21 | 0.5 | 4 | 16 | 16 | 8 | 4 |
| EC22 | 0.5 | 4 | 8 | 8 | 4 | 2 |
| EC23 | 0.25 | 4 | 16 | 4 | 4 | 2 |
| EC24 | 2 | 32 | 128 | 32 | 16 | 8 |
| EC25 | 4 | 32 | 128 | 32 | 16 | 8 |
| EC26 | 2 | 8 | 128 | 32 | 16 | 8 |
| EC27 | 1 | 8 | 128 | 8 | 8 | 4 |
| EC28 | 0.5 | 8 | 32 | 8 | 4 | 4 |
| EC29 | 4 | 32 | 128 | 64 | 32 | 16 |
| EC30 | 4 | 32 | 128 | 64 | 32 | 16 |
| EC31 | 2 | 32 | 128 | 64 | 16 | 32 |
| EC32 | 4 | 32 | 128 | 32 | 16 | 8 |
| EC33 | 2 | 8 | 32 | 8 | 8 | 4 |
| EC34 | 8 | 32 | 128 | 64 | 32 | 16 |
| EC35 | 8 | 64 | 128 | 128 | 64 | 32 |
| EC36 | 4 | 32 | 128 | 64 | 64 | 16 |
| EC37 | 4 | 64 | 128 | 32 | 16 | 8 |
| EC38 | 2 | 16 | 64 | 8 | 8 | 4 |
| EC39 | 8 | 128 | 128 | 64 | 64 | 16 |
| EC40 | 0.5 | 4 | 16 | 16 | 4 | 2 |
| EC41 | 0.5 | 4 | 16 | 16 | 4 | 2 |
| EC42 | 0.25 | 4 | 32 | 8 | 4 | 4 |
| EC43 | 0.5 | 2 | 8 | 4 | 2 | 2 |
| EC44 | 2 | 8 | 32 | 32 | 16 | 8 |
| EC45 | 1 | 32 | 128 | 32 | 16 | 8 |
| EC46 | 2 | 8 | 32 | 32 | 16 | 8 |
| EC47 | 1 | 8 | 16 | 8 | 4 | 2 |
| EC48 | 1 | 16 | 32 | 16 | 8 | 8 |
| EC49 | 4 | 16 | 64 | 128 | 32 | 16 |
| EC50 | 8 | 32 | 256 | 64 | 32 | 16 |
| EC51 | 4 | 32 | 64 | 32 | 16 | 8 |
| EC52 | 4 | 32 | 128 | 16 | 8 | 4 |
| EC53 | 2 | 32 | 128 | 16 | 8 | 4 |
| EC54 | 8 | 64 | 128 | 128 | 32 | 16 |
| EC55 | 8 | 32 | 256 | 8 | 4 | 2 |
| EC56 | 4 | 64 | 128 | 128 | 64 | 16 |
| EC57 | 8 | 64 | 128 | 64 | 16 | 8 |
| EC58 | 4 | 64 | 256 | 32 | 16 | 8 |
| EC59 | 16 | 128 | 256 | 128 | 128 | 32 |
| EC60 | 0.016 | 0.125 | 1 | 0.016 | 0.016 | 0.004 |
| EC61 | 0.25 | 0.5 | 2 | 16 | 16 | 4 |
| EC62 | 0.5 | 4 | 32 | 0.25 | 0.125 | 0.016 |
| EC63 | 1 | 16 | 64 | 0.5 | 0.25 | 0.06 |
| EC64 | 2 | 16 | 128 | 1 | 0.25 | 0.25 |
| EC65 | 8 | 64 | 128 | 16 | 8 | 8 |
| EC66 | 0.5 | 4 | 64 | 8 | 2 | 0.03 |
| EC67 | 1 | 16 | 64 | 1 | 0.25 | 0.125 |
| EC68 | 8 | 64 | 128 | 8 | 0.5 | 0.25 |
| EC69 | 16 | 128 | 256 | 4 | 1 | 0.5 |
| EC70 | 0.016 | 1 | 0.5 | 0.016 | 0.016 | 0.004 |
| EC71 | 0.06 | 2 | 2 | 0.06 | 0.03 | 0.004 |
| EC72 | 0.25 | 2 | 16 | 0.125 | 0.06 | 0.03 |
| EC73 | 1 | 8 | 64 | 0.5 | 0.125 | 0.06 |
| EC74 | 0.5 | 8 | 64 | 0.5 | 0.25 | 0.06 |
| EC75 | 2 | 32 | 128 | 1 | 0.25 | 0.125 |
| EC76 | 0.5 | 4 | 32 | 0.25 | 0.125 | 0.03 |
| EC77 | 0.5 | 16 | 64 | 1 | 0.5 | 0.125 |
| EC78 | 8 | 64 | 128 | 2 | 1 | 0.25 |
| EC79 | 16 | 128 | 256 | 128 | 2 | 0.5 |

Table S3: Modal MICs of ciprofloxacin and delafloxacin according to pH against all strains
